# Supplementary material for: HLA matching or CRISPR editing of HLA class I/II enables engraftment and effective function of allogeneic human regulatory T cell therapy in a humanized mouse transplantation model
Source: Nat Commun. 2025 Oct 13;16:9090. doi: 10.1038/s41467-025-64945-3 (PMC12518618; doi:10.1038/s41467-025-64945-3)
Supplement: Supplementary file 1 — Supplementary Information [file 41467_2025_64945_MOESM1_ESM.pdf]

A

	HLA-A		HLA-B		HLA-DR	
Donor 1	11	1	38	60	1	13
Donor 2	3	2	18	7	15	
Donor 3	24		7		7	11

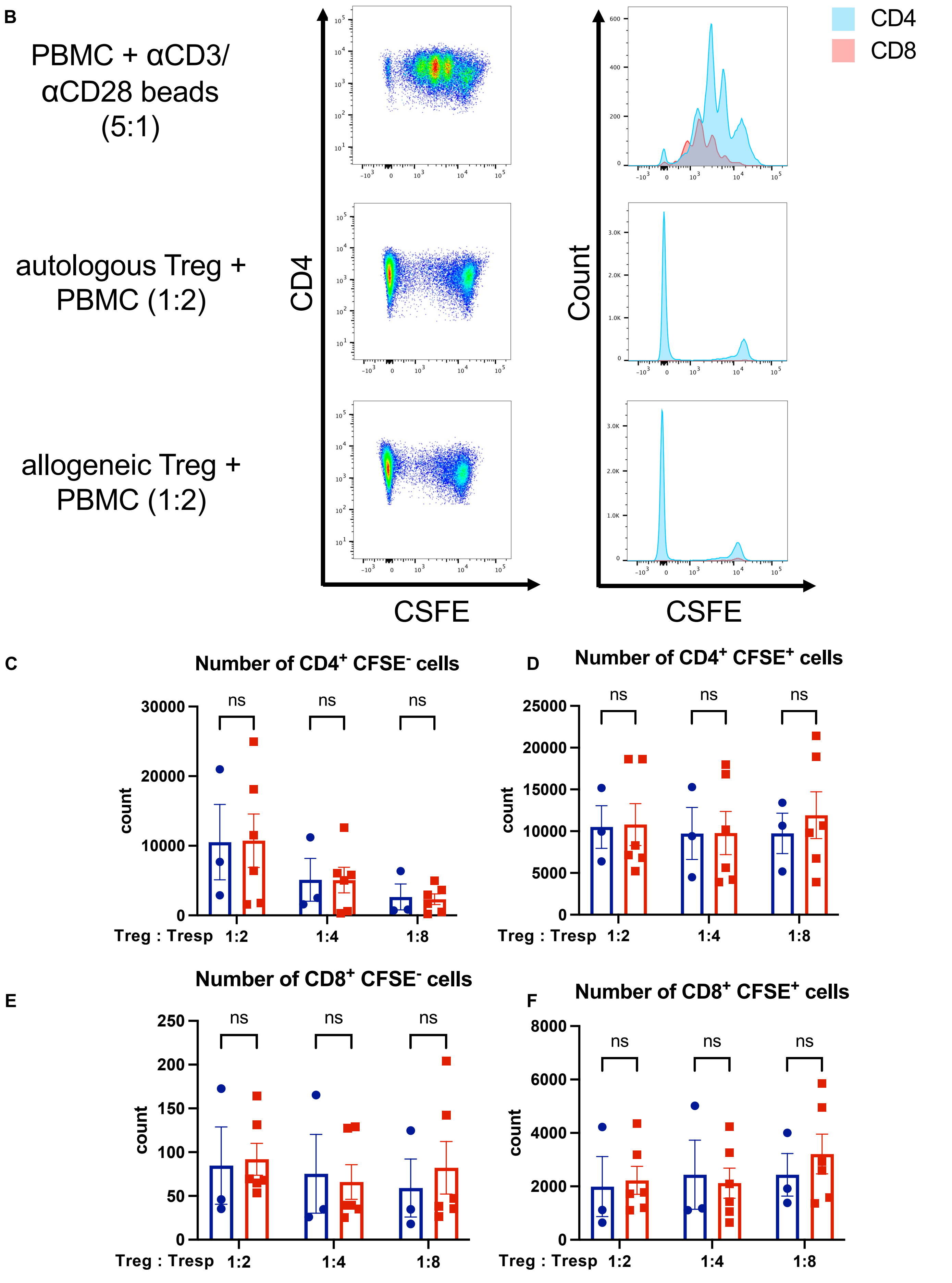

Suppl. Fig. S1. Autologous and allogeneic Tregs suppress responder T cell proliferation equivalently in vitro. (**A**) Donor HLA typing. (**B**) Responder PBMCs were stained with CFSE and cultured at $1x10^5$ per well for 72h with autologous or allogeneic *in vitro*-expanded Treg. αCD3αCD28 T cell activator beads (Thermo Fisher Scientific) were added at a 1:5 bead:cell ratio. Representative flow cytometry dot plots of $CD4^+$ CFSE signal dilution (left column) and histograms of $CD4^+$ and $CD8^+$ CFSE intensity (right column) for each condition. (**C-F**) Absolute numbers of $CD4^+$ and $CD8^+$ recovered from lavage by CFSE stain. Statistical significance for autologous versus allogeneic Tregs, across all Treg:responder ratios, was assessed using two-way repeated measures ANOVA with two-tailed Bonferroni tests for pairwise comparisons. (**C**) $CD4^+$ $CFSE^-$, F(1,6)=0.00019, p=0.09894, n^2p=0.0003, 95%CI=[-8731, 8830] (p>0.05 for all pairs) (**D**) $CD4^+$ $CFSE^+$, F(1,6)=0.6707, p=0.4441, n^2p=0.008, 95%CI=[-3330, 1660] (p>0.05 for all pairs) (**E**) $CD8^+$ $CFSE^-$, F(1,6)=0.6521, p=0.4502, n^2p=0.00425, 95%CI=[-28.14, 14.17] (p>0.05 for all pairs) and (**F**) $CD8^+$ $CFSE^+$ F(1,6)=0.7490, p=0.42, n^2p=0.007, 95%CI=[-886.6, 423.3] (p>0.05 for all pairs) cells. Source data are provided as a Source Data file.

A

	HLA-A		HLA-B		HLA-DR	
Donor 1	11	1	38	60	1	13
Donor 2	24	2	64	62	7	11

B

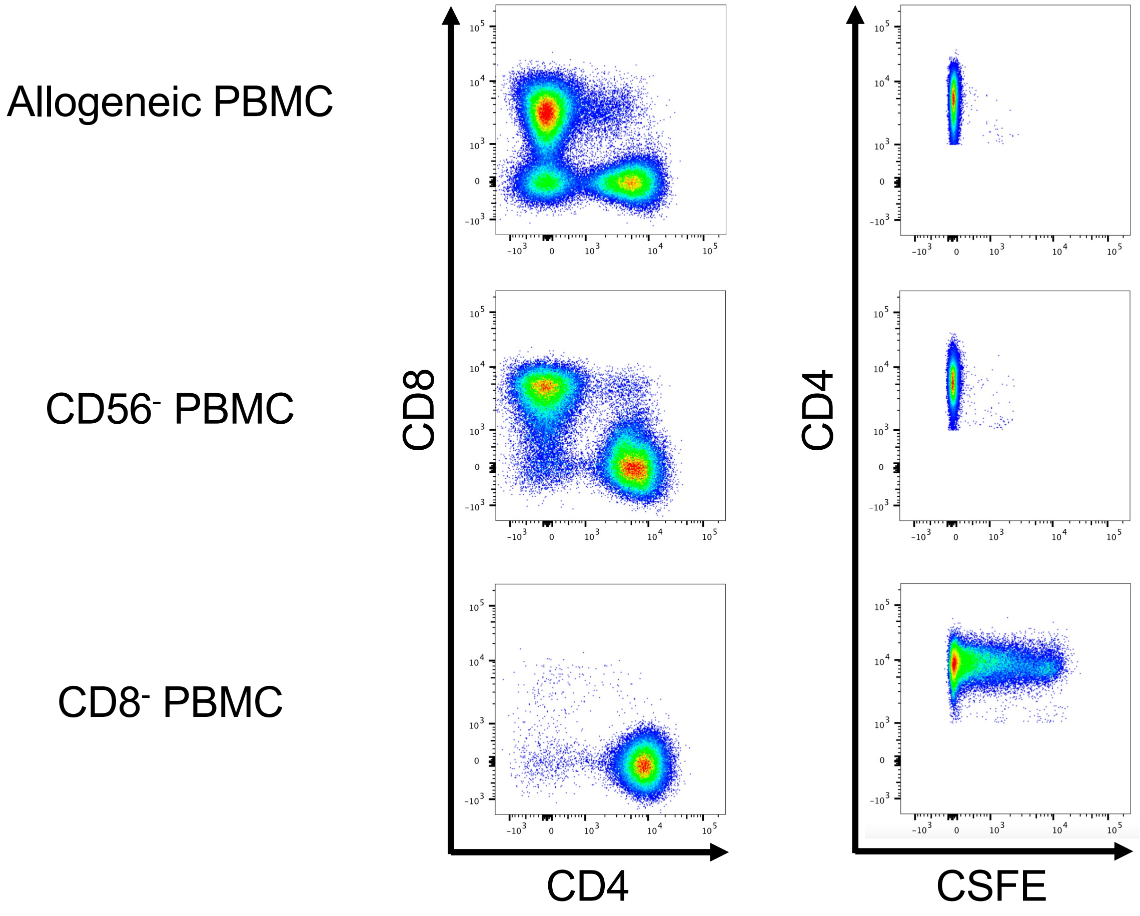

Suppl. Fig. S2. Donor HLA type for Figure 2 and representative flow cytometry plots of CD56- or CD8- depleted PBMC. (**A**) Donor HLA typing. (**B**) BALB/cRag2$^{-/-}$cγc$^{-/-}$ mice received 5x10^{6} CFSE-stained Treg with 5x10^{6} of either whole PBMC, CD8-depleted PBMC, or CD56-depleted PBMC in pure RPMI via intraperitoneal injection. Seven days after injection, cells were recovered from the peritoneal cavity by lavage. Human lymphocytes in the effluent were enumerated and phenotyped by flow cytometry. Representative plots of CD4^{+} and CD8^{+} staining within the hCD45^{+}CD3^{+} live lymphocyte gate (left column) and CFSE staining within hCD45^{+}CD3^{+}CD4^{+} live lymphocyte gate (right column, representing Treg).

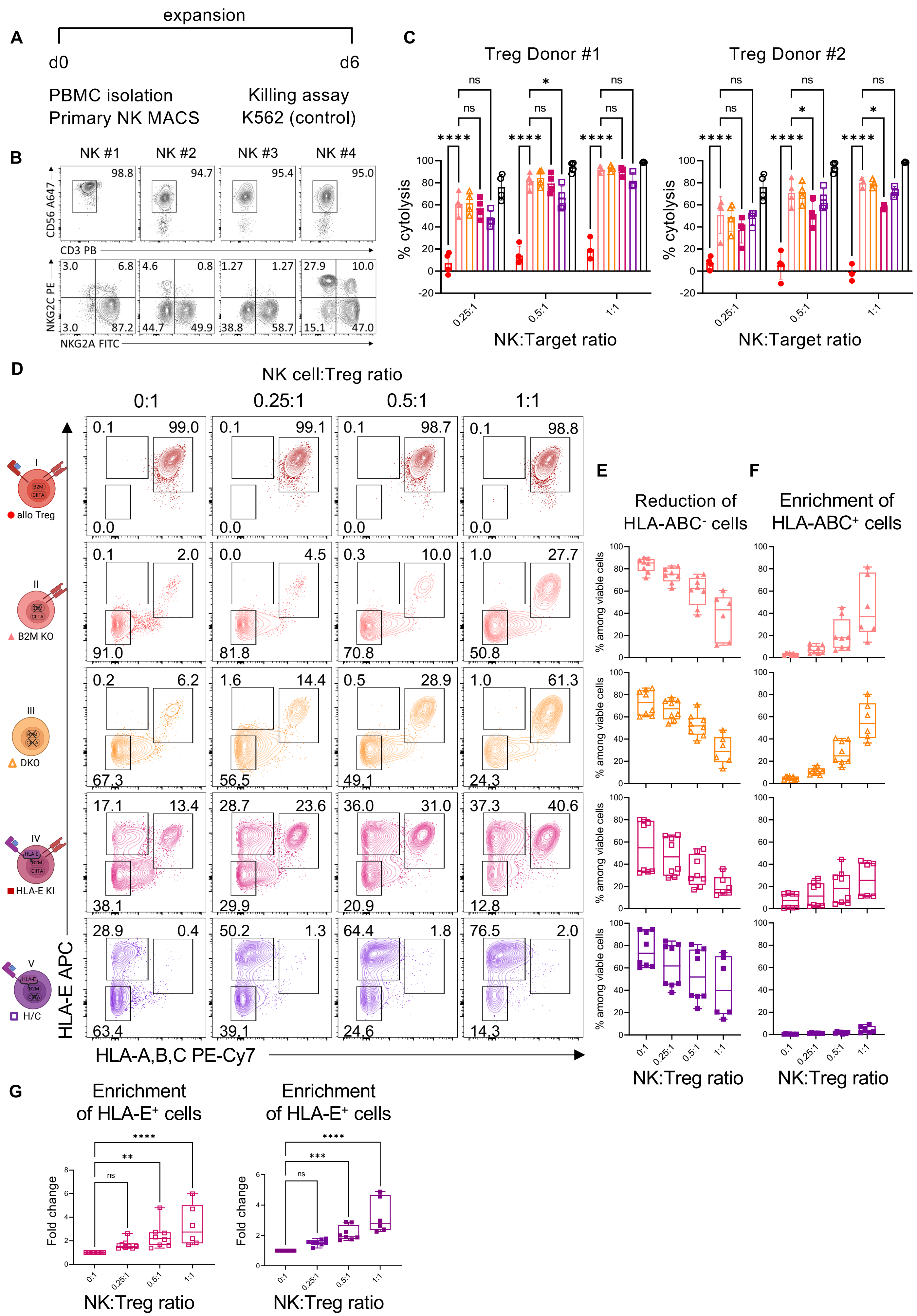

A
expansion
d0
d6
PBMC isolation
Primary NK MACS
Killing assay
K562 (control)
B
NK #1
NK #2
NK #3
NK #4
CD56 A647
CD3 PB
NKG2C PE
NKG2A FITC
C
Treg Donor #1
Treg Donor #2
% cytolysis
NK:Target ratio
D
NK cell:Treg ratio
allo Treg
B2M KO
DKO
HLA-E KI
H/C
HLA-E APC
HLA-A,B,C PE-Cy7
E
Reduction of HLA-ABC- cells
F
Enrichment of HLA-ABC+ cells
% among viable cells
NK:Treg ratio
G
Enrichment of HLA-E+ cells
Fold change
NK:Treg ratio

Suppl. Fig. S3. Co-culture of primary expanded NK cell lines with gene edited Treg demonstrates partial evasion of NK mediated missing-self cytolysis through expression of transgenic HLA-E. (**A**) NK cells were MACS isolated and expanded for 6 days. (**B**) Phenotype of the NK cells isolated from peripheral blood of 4 healthy donors by flow cytometric analysis of CD56 AF647, CD3 PE, NKG2C PE and NKG2A FITC. (**C**) Cytolysis of Tregs and K562 cells by NK cells normalized to target only control after co-culture at NK:target cell ratios of 0.25:1, 0.5:1 and 1:1. (**D**) Representative contour plots of HLA-A,B,C and HLA-E staining upon 20 hours co-culture of primary NK cells and Treg cells at various ratios: 0:1, 0.25:1, 0.5:1, 1:1. NK cells were pre-labelled with CFSE. Red represents alloTreg cells; pink represents B2M KO Treg cells; gold represents DKO Treg cells; magenta represents HLA-E KI Tregs; and purple represents HLA-E KI and CIITA KO (H/C) Tregs. (**E**) Percentage of HLA-A, B, C negative cells among viable cells at various ratios of NK cells to Tregs. N=6-8. (**F**) Percentage of HLA-A, B, C positive cells among viable cells at various ratios of NK cells to Tregs. N=6-8. (**G**) Enrichment of HLA-E positive cells when co-culturing of Tregs with NK cells at various ratios. (N=6-8). For statistical analyses ordinary two-way ANOVAs with main effects only were used to compare each column mean with the control column (C: B2M KO; G: NK:Treg ratio 0:1). Post-hoc two-tailed adjusted Dunnett testing was performed for statistical hypothesis testing (ns: donor 1 (left) – $t(19) = –1.50$, $p = 0.197$, $d = 1.06$, 95% CI = [–1.611, 0.266]; donor 2 (right) – $t(19) = –1.67$, $p = 0.135$, $d = 1.18$, 95% CI = [–1.185, 0.132]; *: $p < 0.05$; **: $t(19) = –3.15$, $p = 0.003$, $d = 2.23$, 95% CI = [–2.350, –0.473]; ***: $t(19) = –3.63$, $p = 0.0008$, $d = 2.57$, 95% CI = [–1.801, –0.484]; ****: donor 1 – (left) $t(19) = –4.46$, $p < 0.0001$, $d = 3.16$, 95% CI = [–3.252, –1.176], donor 2 (right) – $t(19) = –6.41$, $p < 0.0001$, $d = 4.53$, 95% CI = [–2.958, –1.502]). Panel D Created in BioRender. Wagner, D. (2025) https://BioRender.com/ydoiabq. Source data are provided as a Source Data file.

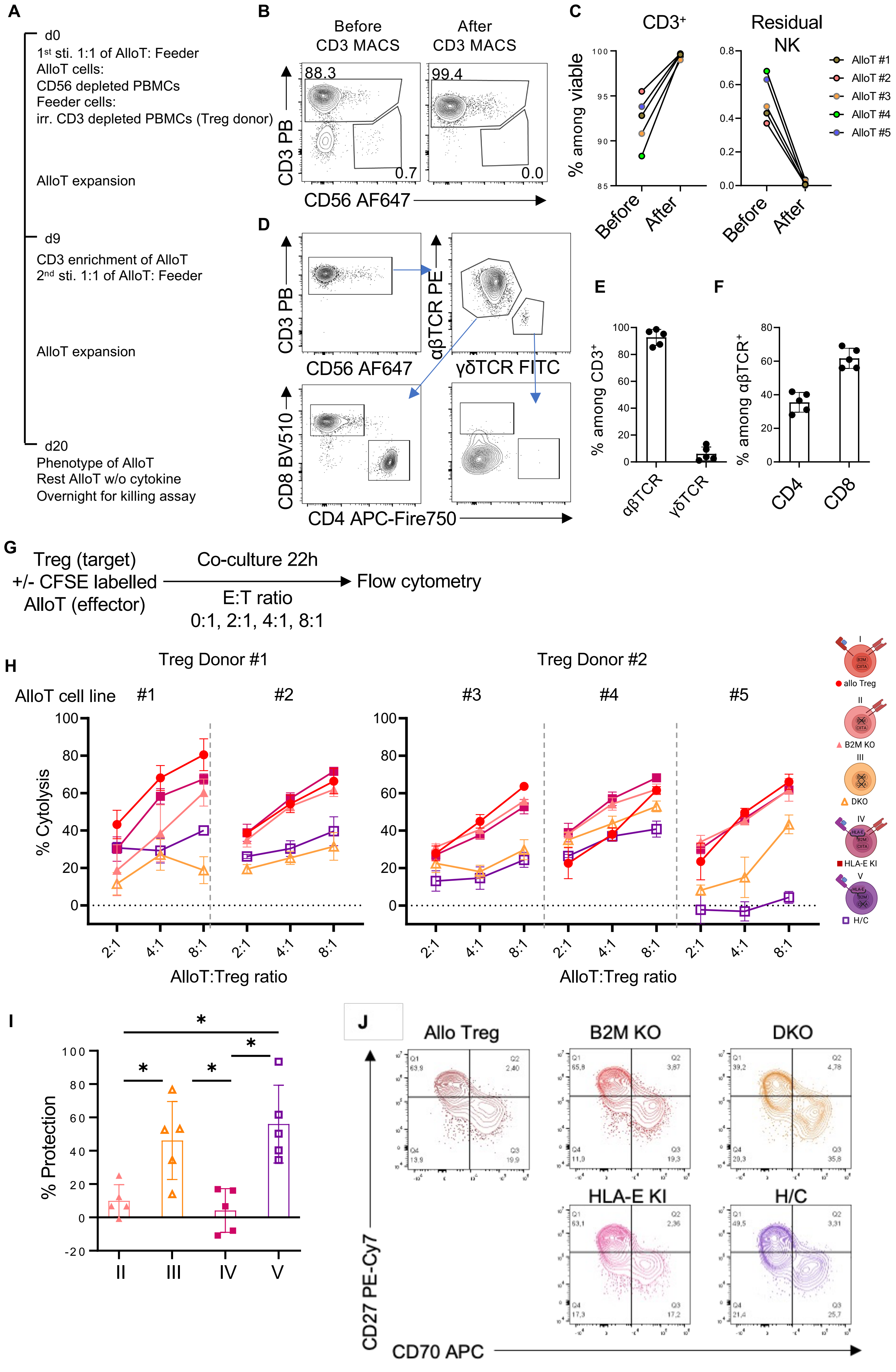

A
d0
1st sti. 1:1 of AlloT: Feeder
AlloT cells:
CD56 depleted PBMCs
Feeder cells:
irr. CD3 depleted PBMCs (Treg donor)
AlloT expansion
d9
CD3 enrichment of AlloT
2nd sti. 1:1 of AlloT: Feeder
AlloT expansion
d20
Phenotype of AlloT
Rest AlloT w/o cytokine
Overnight for killing assay
B
Before CD3 MACS
After CD3 MACS
88.3
0.7
99.4
0.0
CD3 PB
CD56 AF647
C
CD3+
Residual NK
% among viable
Before
After
AlloT #1
AlloT #2
AlloT #3
AlloT #4
AlloT #5
D
CD3 PB
CD56 AF647
αβTCR PE
γδTCR FITC
CD8 BV510
CD4 APC-Fire750
E
F
% among CD3+
αβTCR
γδTCR
% among αβTCR+
CD4
CD8
G
Treg (target)
+/- CFSE labelled
AlloT (effector)
Co-culture 22h
E:T ratio
0:1, 2:1, 4:1, 8:1
Flow cytometry
H
Treg Donor #1
Treg Donor #2
AlloT cell line #1 #2 #3 #4 #5
% Cytolysis
AlloT:Treg ratio
allo Treg
B2M KO
DKO
HLA-E KI
H/C
I
% Protection
II III IV V
J
Allo Treg
B2M KO
DKO
HLA-E KI
H/C
CD27 PE-Cy7
CD70 APC

Suppl. Fig. S4. Editing of HLA Class I and II protects Tregs from allogeneic T cell killing. **(A)** Allogeneic T cells were generated over three weeks with two rounds of stimulation by irradiated (30Gy) CD3-depleted PBMCs from the Treg donor prior to killing assay. **(B)** Representative contour plots of CD56 AF647 and CD3 PB staining of the allogenic T cells before and after CD3 MACS enrichment at day 9 prior to the second round of stimulation. **(C)** Percentages of $CD3^+$ T cells (left) and of residual $CD56^+CD3^-$ NK cells (right) of five allogenic T cells before and after CD3 MACS enrichment (n=5). **(D-F)** Allogenic T cell phenotype one day prior to killing assay. **(D)** Representative contour plots of CD3 PB, CD56 AF647, αβTCR PE, γδTCR FITC, CD4 APC-Fire750, CD8 BV510. **(E)** Percentages of αβTCR and γδTCR amongst $CD3^+$ T cells (n=5). **(F)** Percentages of $CD4^+$ and $CD8^+$ lymphocytes amongst $\alpha\beta TCR^+$ T cells (n=5). **(G)** Schematic overview of the allogenic T cell killing assay. Allogeneic T cells (effector, E) were pre-labelled with CFSE prior to co-culturing with Tregs (target, T) at E:T ratios of 0:1, 0.25:1, 0.5:1, 1:1 for 22 hours. **(H)** Cytolysis of allogeneic Treg cells with different gene editing strategies from two donors by allo-specific T cell lines. Each dot represents mean of three technical replicates. Percentage cytolysis was normalized to cell counts from Treg cells alone (without allo-specific T cells) = E:T ratio 0:1. (**I**) Relative protection of different gene edited Treg products in comparison to unedited allogeneic Treg at 8:1 E:T ratio combining all data from the five experiments shown in H. Percentage protection represents the number of surviving modified Treg relative to wild-type Tregs, expressed as a percentage. For statistical analysis one-way ANOVA with Geisser-Greenhouse correction was performed. To adjust for multiple corrections, two-tailed follow up testing was performed with the Tukey test (ns: $p \geq 0.05$; *: $p < 0.05$ **: $p < 0.01$). Only statistically significant results are indicated. II-III: $t(12) = -2.92$, $p = 0.019$, $d = 2.06$, 95% CI = [–63.17, –9.19]; II-V: $t(12) = -2.26$, $p = 0.045$, $d = 1.60$, 95% CI = [–90.47, –1.63]; III-IV: $t(12) = 2.89$, $p = 0.019$, $d = 2.04$, 95% CI = [10.28, 73.67]; IV-V: $t(12) = -3.06$, $p = 0.016$, $d = 2.16$, 95% CI = [–88.76, –14.92].(**J**) Flow cytometry plots depicting CD27 and CD70 expression after Treg expansion within each group. Panel H created in BioRender. Wagner, D. (2025) https://BioRender.com/ydoiabq. Source data are provided as a Source Data file.

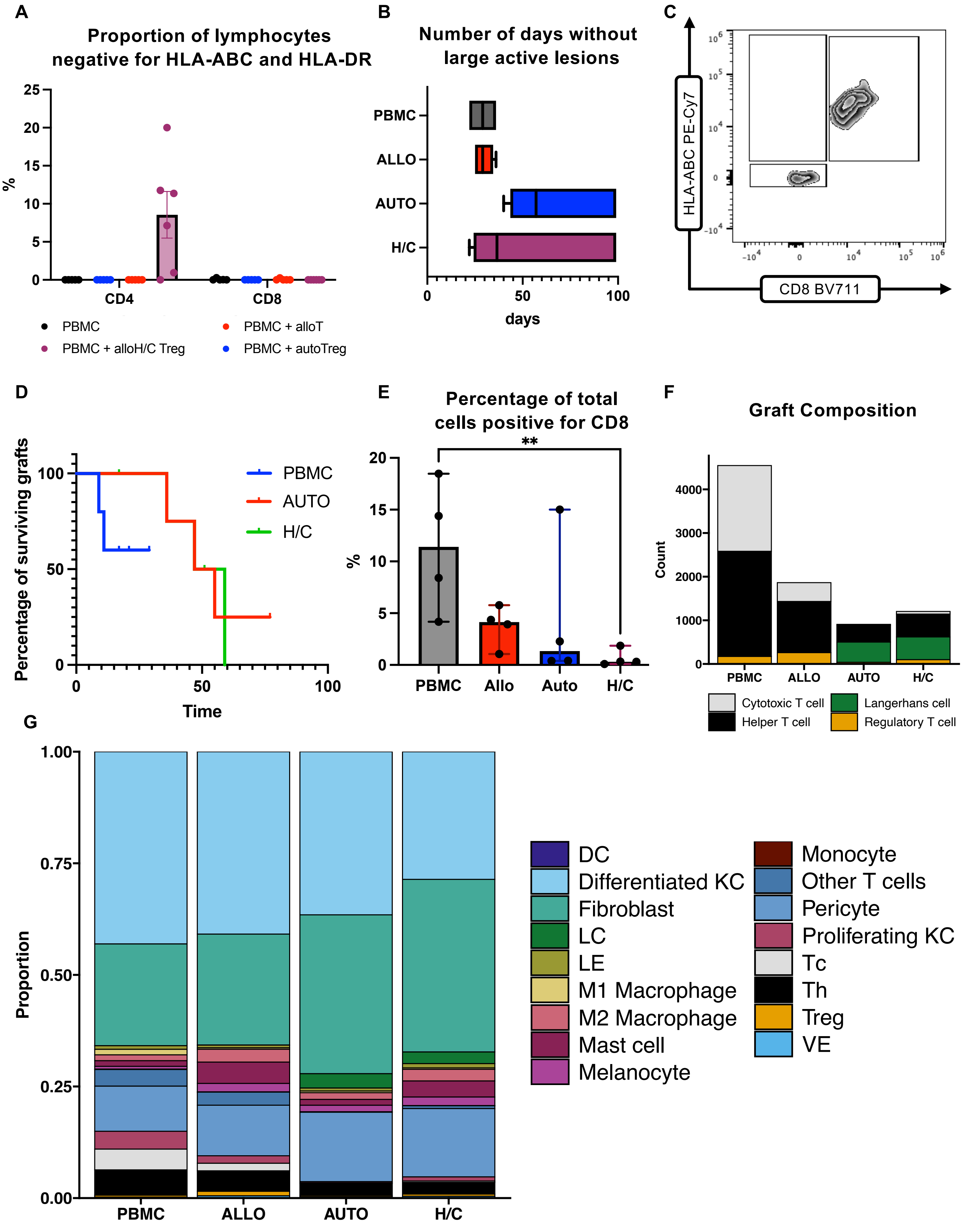

A
Proportion of lymphocytes negative for HLA-ABC and HLA-DR
%
CD4
CD8
PBMC
PBMC + alloT
PBMC + alloH/C Treg
PBMC + autoTreg
B
Number of days without large active lesions
PBMC
ALLO
AUTO
H/C
days
C
HLA-ABC PE-Cy7
CD8 BV711
D
Percentage of surviving grafts
PBMC
AUTO
H/C
Time
E
Percentage of total cells positive for CD8
**
%
PBMC
Allo
Auto
H/C
F
Graft Composition
Count
PBMC
ALLO
AUTO
H/C
Cytotoxic T cell
Langerhans cell
Helper T cell
Regulatory T cell
G
Proportion
PBMC
ALLO
AUTO
H/C
DC
Differentiated KC
Fibroblast
LC
LE
M1 Macrophage
M2 Macrophage
Mast cell
Melanocyte
Monocyte
Other T cells
Pericyte
Proliferating KC
Tc
Th
Treg
VE

Suppl. Fig. S5. HLA-edited Tregs persist in circulation and minimize CD8+ infiltration into skin allografts. (**A**) Quantification of HLA-DR and HLA-ABC staining on human lymphocytes within peripheral blood of mice 21 days following adoptive transfer. (**B**) Number of days before the development of significant rejection by experimental group (Kruskal-Wallis non-parametric ANOVA: H(3)=8.670, p = 0.0340, ϵ^2=0.33. Post-hoc groupwise two-tailed Dunn's tests: allogeneic versus autologous Treg Z=2.441, p=0.0147, r^2=0.6; allogeneic versus H/C Treg Z=1.038, p = 0.2995, r^2=0.1; autologous versus H/C Treg Z=1.512, p=0.1306, r^2=0.21). (**C**) Representative gating of HLA-ABC positive and negative events. Upstream gating is: singlets > lymphocyte size selection > viability > hCD45 > OR gate on CD4+ and CD8+. (D–**G**) Immunodeficient BALB/c Rag2$^{-/-}$ cγc$^{-/-}$ mice grafted with human skin received intraperitoneal human PBMCs (5x10^6) either alone or with autologous, allogeneic, or H/C edited Tregs (1 x 10^6). Grafts were monitored for macroscopic signs of rejection and were harvested at 15 days following adoptive transfer. (**D**) A replicate of the skin graft experiment presented in Figure 5B. This experiment coincided with a move to a new animal facility, where we observe a markedly higher background incidence of xenograft-versus-host disease (XVHD), confounding the interpretation of long-term graft survival, although H/C Treg produce a similar enhancement in graft survival as autologous Tregs.A replicate of the skin graft experiment presented in Figure 5B. This experiment coincided with a move to a new animal facility, where we observe a markedly higher background incidence of xenograft-versus-host disease (XVHD), confounding the interpretation of long-term graft survival. (**E**) Proportion of total cells positive for CD8 within skin grafts measured by immunohistochemistry. Groupwise differences assessed with the Kruskal-Wallis test against the reference group of PBMC (H(3)=9.066, p=0.0120, ϵ^2=0.0506). Post-hoc groupwise two-tailed Dunn's tests: (allo) Z=1.114, p=0.7959, r=0.394; (auto) Z=1.559, p=0.3566, r=0.551; (H/C) Z=2.970, p=0.0089, r≈0.99 (**F**) Compositional analysis of skin grafts restricted to T and Langerhans cell subsets identified by *in situ* gene expression. (**G**) Total cellular composition of skin grafts at harvest (DC: dendritic cell, LC: Langerhans cell, LE: lymphatic endothelium, Tc: cytotoxic T cell, Th: helper T cell, Treg: regulatory T cell, VE: vascular endothelium) identified by *in situ* gene expression. Source data are provided as a Source Data file.
