# Supplementary material for: HLA matching or CRISPR editing of HLA class I/II enables engraftment and effective function of allogeneic human regulatory T cell therapy in a humanized mouse transplantation model
Source: Nat Commun. 2025 Oct 13;16:9090. doi: 10.1038/s41467-025-64945-3 (PMC12518618; doi:10.1038/s41467-025-64945-3)
Supplement: Supplementary file 2 — Description of Additional Supplementary Files [file 41467_2025_64945_MOESM2_ESM.pdf]

Title: Supplementary Data 1

Description: HLA typing of PBMC and Treg donor pairs for Figure 3. PBMCs and Tregs were HLA�typed by the Oxford Transplant Centre Histocompatibility and Genetics Laboratory. HLA loci for all experimental pairings are presented for each HLA allele. For each allele, the number of mismatches are quantified. The B130/B209 pair is completely mismatched at HLA-A,-B,-C,-DR (1,2,1,2); B218/B209 is partially mismatched at these loci (0,1,1,2); and B208/B150 is partially matched (0,0,0,1).

Title: Supplementary Data 2

Description: Homology-Directed DNA Repair Template (HDRT) used for HLA-E knock-in into B2M locus

Title: Supplementary Data 3

Description: CRISPR-Cas9 guide RNAs used for editing of B2M and CIIT
