# Supplementary material for: HLA matching or CRISPR editing of HLA class I/II enables engraftment and effective function of allogeneic human regulatory T cell therapy in a humanized mouse transplantation model
Source: Nat Commun. 2025 Oct 13;16:9090. doi: 10.1038/s41467-025-64945-3 (PMC12518618; doi:10.1038/s41467-025-64945-3)
Supplement: Supplementary file 4 — Reporting Summary [file 41467_2025_64945_MOESM4_ESM.pdf]

nature portfolio

Corresponding author(s): Fadi Issa, Joanna Hester, Dimitrios Wagner

Last updated by author(s): May 9, 2025

Reporting Summary

Nature Portfolio wishes to improve the reproducibility of the work that we publish. This form provides structure for consistency and transparency in reporting. For further information on Nature Portfolio policies, see our Editorial Policies and the Editorial Policy Checklist.

Statistics

For all statistical analyses, confirm that the following items are present in the figure legend, table legend, main text, or Methods section.

n/a	Confirmed	
☐	☒	The exact sample size (*n*) for each experimental group/condition, given as a discrete number and unit of measurement
☐	☒	A statement on whether measurements were taken from distinct samples or whether the same sample was measured repeatedly
☐	☒	The statistical test(s) used AND whether they are one- or two-sided<br>*Only common tests should be described solely by name; describe more complex techniques in the Methods section.*
☐	☒	A description of all covariates tested
☐	☒	A description of any assumptions or corrections, such as tests of normality and adjustment for multiple comparisons
☐	☒	A full description of the statistical parameters including central tendency (e.g. means) or other basic estimates (e.g. regression coefficient) AND variation (e.g. standard deviation) or associated estimates of uncertainty (e.g. confidence intervals)
☐	☒	For null hypothesis testing, the test statistic (e.g. *F*, *t*, *r*) with confidence intervals, effect sizes, degrees of freedom and *P* value noted<br>*Give P values as exact values whenever suitable.*
☒	☐	For Bayesian analysis, information on the choice of priors and Markov chain Monte Carlo settings
☒	☐	For hierarchical and complex designs, identification of the appropriate level for tests and full reporting of outcomes
☐	☒	Estimates of effect sizes (e.g. Cohen's *d*, Pearson's *r*), indicating how they were calculated

Our web collection on statistics for biologists contains articles on many of the points above.

Software and code

Policy information about availability of computer code

Data collection	Spatial Transcriptomic analyses were performed on the Xenium Analyser (10X Genomics) running onboard analysis software version 3.0. No additional software was utilized in the collection of data for this study.
Data analysis	GraphPad Prism version 9.0 or version 10.3.1 for MacOS (GraphPad Software, La Jolla California USA) was used for data analysis and graph production. Differential gene expression was performed with DESeq2 (version 1.46.0) on pseudobulks of grafts stratified by damage and visualized with pheatmap (version 1.0.12). Count matrices were processed in Seurat (version 5.1.0). Spatial transcriptomics data were processed with SpatialData (version 0.1.2) and NapariViewer (version 0.4.1). Sequencing reads were aligned and evaluated using the Bismark package (version 0.24.2)

For manuscripts utilizing custom algorithms or software that are central to the research but not yet described in published literature, software must be made available to editors and reviewers. We strongly encourage code deposition in a community repository (e.g. GitHub). See the Nature Portfolio guidelines for submitting code & software for further information.

Data

Policy information about availability of data

All manuscripts must include a data availability statement. This statement should provide the following information, where applicable:

- Accession codes, unique identifiers, or web links for publicly available datasets
- A description of any restrictions on data availability
- For clinical datasets or third party data, please ensure that the statement adheres to our policy

All data are presented in the main text or the supplementary materials. Spatial transcriptomic datasets (Fig. 5 and Suppl. Fig. 5) are publicly available on FigShare (DOI: 10.6084/m9.figshare.28988606) at the time of publication. Source Data are provided with this manuscript.

Research involving human participants, their data, or biological material

Policy information about studies with human participants or human data. See also policy information about sex, gender (identity/presentation), and sexual orientation and race, ethnicity and racism.

Reporting on sex and gender	Human skin was procured from female donors due to the nature of compatible surgery. Human PBMCs utilised within the study were donated by healthy anonymous donors and therefore sex and gender data are not available.
Reporting on race, ethnicity, or other socially relevant groupings	All biological material used within the study were derived from anonymous donors and therefore no demographic data are available.
Population characteristics	All biological material used within the study were derived from anonymous donors and therefore no population characteristic data are available.
Recruitment	Patients undergoing compatible surgical procedures were recruited and gave full informed written consent to provide material that would otherwise be discarded during surgery. Human peripheral blood mononuclear cells (PBMC) were isolated from the blood of healthy donors (provided by the National Blood Service, Oxford, UK) or was obtained after informed and written consent (Charité ethics committee approval EA4/091/19)
Ethics oversight	Human skin was procured with full informed written consent and with ethical approval from the Oxfordshire Research Ethics Committee (REC B), study number 07/H0605/130.

Note that full information on the approval of the study protocol must also be provided in the manuscript.

Field-specific reporting

Please select the one below that is the best fit for your research. If you are not sure, read the appropriate sections before making your selection.

☒ Life sciences ☐ Behavioural & social sciences ☐ Ecological, evolutionary & environmental sciences

For a reference copy of the document with all sections, see nature.com/documents/nr-reporting-summary-flat.pdf

Life sciences study design

All studies must disclose on these points even when the disclosure is negative.

Sample size	Sample sizes were based on preliminary results and prior experience with the employed models. For in vitro experiments all conditions were performed in triplicate with the mean value plotted as described in the text. The animals utilised balanced providing robust data without using excessive numbers in accordance with NC3R principles.
Data exclusions	Animal censoring is described within the text and was performed if signs of xenoGvHD developed.
Replication	Findings presented within the study were replicated across several independent experiments using orthogonal complimentary modalities. In vivo experiments were repeated as described in the text.
Randomization	Mice were randomized across experimental groups and cages. Donation of PBMC and human tissue was provided by random donors, with specific HLA pairings identified post hoc as described in the text.
Blinding	Investigators were blinded to experimental group allocation for in vivo experiments. Blinding was not possible during in vitro experiments as each individual experiment was performed by an individual scientist to minimize inter-operator variation.

Reporting for specific materials, systems and methods

We require information from authors about some types of materials, experimental systems and methods used in many studies. Here, indicate whether each material, system or method listed is relevant to your study. If you are not sure if a list item applies to your research, read the appropriate section before selecting a response.

Materials & experimental systems

n/a	Involved in the study	
☐	☒	Antibodies
☒	☐	Eukaryotic cell lines
☒	☐	Palaeontology and archaeology
☐	☒	Animals and other organisms
☒	☐	Clinical data
☒	☐	Dual use research of concern
☒	☐	Plants

Methods

n/a	Involved in the study	
☒	☐	ChIP-seq
☐	☒	Flow cytometry
☒	☐	MRI-based neuroimaging

Antibodies

Antibodies used

The following antibodies (supplier, clone) were used in the study: mouse anti-human CD127 PE (BD, hIL-7R-M21), mouse anti-human CD25 PE-Cy7 (BD, M-A251), mouse anti-human CD3 eFluor450 (Affymetrix, OKT3), mouse anti-human CD4 ECD (Beckmann Coulter, SFCI12T4D11), mouse anti-human CD45 APC (BD, RPA-T4), mouse anti-human CD45 APC (Affymetrix, H130), mouse anti-human CD8 FITC (Affymetrix, SK1), mouse anti-human CD8 PE (BD, HIT8a), mouse anti-human CD8 APC-Cy7 (BD, SK1), rat anti-human FOXP3 FITC (Affymetrix, PCH101), rat anti-human FOXP3 PE (Affymetrix, PCH101), rat anti-human FOXP3 eFluor450 (Affymetrix, PCH101), rat anti-mouse CD45 PE (Affymetrix, 30F11), mouse anti-human HLA-A,B,C PE-Cy7 (BioLegend, W6/32), mouse anti-human HLA-E APC (BioLegend, 3D12), mouse anti-human HLA-DR,DP,DQ FITC (BioLegend, Tü39), mouse anti-human FOXP3 FITC (BD Pharmingen, 259D/C7), mouse anti-human CD25 PC7 (Beckman, B1.49.9), mouse anti-human TNFα A700 (BioLegend, Mab11), mouse anti-human IFNγ APC-eF780 (Invitrogen, 4S.B3), rat IL-2 PE-Cy7 (BioLegend, MQ1-17H12), mouse anti-human CD4 PE (Beckman, 13B8.2), mouse anti-human CD8 PE-Cy7 (BD Pharmingen, RPA-T8), mouse anti-human CD3 PB (BioLegend, UCHT1), mouse anti-human CD56 A647 (BioLegend, 5.1H11), recombinant human anti-human NKG2A FITC (Miltenyi, REA110), recombinant human anti-human NKG2C PE (Miltenyi, REA205), mouse anti-human CD4 PE-eF610 (Invitrogen, RPA-T4), rat anti-mouse CD45 eF450 (Invitrogen 30-F11), mouse anti-human CD3 BV605 (BioLegend, OKT3), mouse anti-human HLA-DR BV650 (BioLegend, L243), mouse anti-human CD8 BV711 (BioLegend, SK1).

Validation

Commercially available antibodies were validated by their respective manufacturers. No additional validation was performed.

Animals and other research organisms

Policy information about studies involving animals; ARRIVE guidelines recommended for reporting animal research, and Sex and Gender in Research

Laboratory animals

BALB/cRag2-/-γc-/- mice (Jackson Laboratory, Bar Harbor, ME, USA) were utilised in this study. Female mice aged between 6 and 24 weeks were were housed in individually ventilated cages in the John Radcliffe Hospital Biomedical Services Unit under specific pathogen-free conditions.

Wild animals

The study did not involve wild animals

Reporting on sex

Animal sex was determined by Jackson Laboratory and confirmed by John Radcliffe Hospital Biomedical Services Unit technical staff. Female age-matched mice were used in this study.

Field-collected samples

The study did not involve samples collected from the field.

Ethics oversight

All protocols were conducted in accordance with the UK Animals (Scientific Procedures) Act (1986) and approved by Oxford University's Committee on Animal Care and Ethical Review.

Note that full information on the approval of the study protocol must also be provided in the manuscript.

Plants

Seed stocks

N/A

Novel plant genotypes

N/A

Authentication

N/A

Flow Cytometry

Plots

Confirm that:

- [x] The axis labels state the marker and fluorochrome used (e.g. CD4-FITC).
- [x] The axis scales are clearly visible. Include numbers along axes only for bottom left plot of group (a 'group' is an analysis of identical markers).
- [x] All plots are contour plots with outliers or pseudocolor plots.
- [x] A numerical value for number of cells or percentage (with statistics) is provided.

Methodology

Sample preparation	Aliquots of cells were taken for staining as described in the text. Briefly, cells were stained in 96-well U bottom plates or directly in FACS tubes. After washing with cold PBS, cell surface staining was performed by incubating primary-conjugated antibodies at 4oC for 20 minutes. For intracellular stains, cells were fixed and permeabilized with eBioscience Foxp3 / Transcription Factor Staining Buffer Set as per the manufacturer's instructions. Non-specific binding was blocked with 2% normal mouse or rat serum before incubation with primary-conjugated antibodies overnight at room temperature. Fluorescence was quantified directly after staining.
Instrument	Fluorescence was measured using BD FACSCanto, Beckman Coulter CytoFLEX, or ThermoFisher Attune NxT flow cytometers.
Software	On board acquisition software from the manufacturer was utilized to generate .fcs files which were then imported into FlowJo (BD Biosciences, version 10.10) for downstream analysis.
Cell population abundance	Upstream gating strategies are described within the text alongside quality assurance data of post-sort purity.
Gating strategy	Gating strategies are described within the text and figure legends. Positive gates were either identified when two clearly distinguishable populations were present and/or were set on appropriate negative isotype or fluorescence-minus-one controls.

- [x] Tick this box to confirm that a figure exemplifying the gating strategy is provided in the Supplementary Information.
